# Introducing an integrated approach for fire safety assessment in healthcare facilities by interval valued neutrosophic-AHP and Fuzzy Inference System

**DOI:** 10.1016/j.heliyon.2025.e41660

**Published:** 2025-01-02

**Authors:** Samaneh Salari, Ali Karimi

**Affiliations:** Department of Occupational Health Engineering, School of Public Health, Tehran University of Medical Sciences, Tehran, Iran

**Keywords:** Fire risk assessment, AHP, Hospital, Healthcare, FIS, Delphi technique, Fire risk, Multi-criteria decision-making

## Abstract

Fire safety in healthcare facilities is extremely important due to limited evacuation capacity of occupants. Therefore, poor fire safety precautions lead to more fatalities and financial losses. This study introduces an effective fire risk management approach for healthcare buildings utilizing an interval valued neutrosophic-fuzzy framework. This framework identifies fire risks and determine appropriate safety measures. In addition, essential fire risk criteria in healthcare centers were systematically identified through a multi-factor decision-making process, employing the Fuzzy-Delphi technique and a comprehensive literature review. The proposed framework initially determines the importance weights of these factors using the Interval Valued Neutrosophic-Analytical Hierarchy Process (IVN-AHP). Subsequently, three main parameters – Potential Risk Level (PRL), Acceptance Risk Level (ARL) and Protection Level (PL) – were calculated as alternatives in IVN-AHP technique using novel mathematical equations. A fuzzy inference system (FIS) was then employed to estimate the risk magnitude and subsequently classify departments within the healthcare building based on their risk level. The fire risk control strategy is then determined based on this risk classification. The proposed approach was applied to departments within a hospital in Iran. Its validity was evaluated by comparing the results with the comprehensive Fire Risk Assessment Method for Engineering (FRAME) technique. Additionally, the sensitivity of the IVN-AHP in determining the weights of factors and alternatives was validated using real data. The results revealed that the utilities and kitchen departments exhibited a critical risk class exceeding 50 %. Furthermore, the operating room, laundry, post-NICU 1 and 2, and waste disposal were classified as critical, with more than 50 % falling within the major risk class. A strong correlation coefficient of 99.1 % was observed between the Fire Risk Magnitude (FRM) obtained using the proposed farmwork and the FRM for occupants obtained using the FRAME technique. These findings demonstrate the applicability and reliability of the proposed approach as a valuable tool for risk management and decision-making in healthcare facilities.

## 1 Introduction

Fires in healthcare facilities occur startling frequently and can have devastating consequences worldwide [1]. Since the outbreak of the Covid-19 pandemic, hospital fires have caused over 250 deaths in several countries [2,3]. According to the NFPA, poor fire safety in healthcare facilities has led to hundreds of death in developing countries [1,3,4].

Healthcare facilities possess distinctive characteristics that influence fire safety, setting them apart from other building types [1,5,6]. Therefore, these characteristics should be considered when assessing the fire risk of these places.

For instance, in 2024, the Gandhi Hospital in Iran experienced a widespread fire caused by an electrical wire short circuit and a high fire load. On the other hand, a medical gas leak and short-circuit in operating room of the Sina medical center in 2020 resulted in an explosion that led to the 19 deaths and many injuries [7]. Furthermore, despite the high level of fire safety in the United States, approximately 1100 fires are reported to fire departments annually, reported to the NFPA [8]. Therefore, healthcare centers should have a comprehensive and precise fire safety management strategy.

While checklists, codes, and standards specific to healthcare facilities are examples of qualitative procedures [9,10], Semi-quantitative fire risk assessment techniques include GRETENER [11], FRAME, CFSES, and FRIME, which are applicable to all types of building used for healthcare centers [9,12]. However, several studies [[13], [14], [15], [16], [17], [18]] employed different disambiguation methodologies for risk assessment because of the ambiguity and inconsistency in expert opinions and uncertainty regarding the nature of risk assessment.

On the other hand, the variables and parameters affecting the fire safety of healthcare facilities must be considered for a quantitative fire risk assessment to be realistic and accurate [19]. For this reason, Multi-Criteria Decision-Making (MCDM) techniques have been applied recently in a number of studies to assess the risk associated with hospitals and medical facilities [20,21].

In this study, parameters of potential risk level (PRL), risk acceptance level (RAL) and protection level (PL) were quantified by using a combination of MCDM techniques. This new integrated approach can be used to analyze fire safety for healthcare facilities. In addition, Fire preventive measures are determined based on the risk magnitude obtained from the fuzzy inference system.

### 1.1 Advantages and novelty of this study

The advantages of this study and the research gaps are listed below.

1. We reviewed literature and the codes, standards, and guidelines pertaining to healthcare facilities, 42 factors were discovered. Following that, the most crucial criteria in fire risk assessment in healthcare centers classified and selected using the F-Delphi technique. These parameters can be used in future studies to model fire risk assessment.
2. One of the main problems in risk assessment is dealing with the related uncertainty inherent in its nature and in expert opinions. This issue has been investigated in several studies. Despite the significance of healthcare fire safety assessment, a few studies have been conducted regarding this issue. There are various strategies to overcome uncertainties, including machine learning [22,23], fuzzy logic [24], and probabilistic models [25]. Neutrosophic theory was created by Smarandach in 1995 as an approach of effectively and efficiently handling inconsistent, uncertain, and ambiguous information [26]. In addition, fuzzy logic assigns uncertainty to the truth and falsity membership variables, whereas Neutrosophic logic provides a new parameter termed "uncertainty" and more accurately represents ambiguity than fuzzy logic [27].
3. Experts have more freedom to express their opinions and judgments when using interval-valued neutrosophic scales of linguistic terms. Moreover, the weight of elements influencing fire safety can be estimated using the IVN-AHP with a high degree of accuracy and reliability.
4. The IVN-AHP alternatives in this study include PRL, RAL and PL. By using the F-Delphi technique to choose candidate factors, we have quantified these parameters in a novel way. The user could analyze the fire safety for the department being investigated based on the level of the three parameters.
5. The risk magnitude is estimated in the fuzzy inference system after computing the normalized weight of these three parameters. The required fire preventative measures are determined based on the risk magnitude, which is a crucial action in fire risk management.

## 2 Literature review

AHP technique has been frequently utilized in numerous literatures [[28], [29], [30]]. For example, the study by Gul et al. uses a two-stage fuzzy multi-criteria technique to estimate risks for hospitals. The Fuzzy Analytic Hierarchy Process (FAHP) was used to weigh five risk parameters, while the fuzzy VIKOR (FVIKOR) approach was applied for prioritizing hazard types in each department [29]. Badida et al., 's 2023 study highlights the significance of an occupational health and safety policy to lower workplace accidents. The authors employed fuzzy multi-criteria decision-making methods in a Chennai hospital, combining FAHP and TOPSIS techniques. Electrical hazards improper medical equipment and air conditioning hazards were the top three hazards identified [31].

Furthermore, these MCDM techniques have been applied recently in various research projects for various objectives related to risk assessment in healthcare facilities. For instance, they have been applied to predict the performance of a hospital information system, manage the security risk of a healthcare web application, and to find notable deviations in the oxygen supply system.

Additionally, these MCDM techniques have been used recently in a number of research projects with a variety of risk assessment-related goals for healthcare facilities [[32], [33], [34], [35]]. They have been used, for example, to identify significant variations in the oxygen supply system [36] and to predict the performance of a hospital information system [28] and manage the security risk of a healthcare web application [37].

On the other hand, due to the fact that conventional FMEA is insufficient for evaluating failure modes, weighing risk factors, and assessing failure modes. Different models were developed for risk assessment and analysis in different studies to address the aforementioned issues. Various studies in this field are listed in Table 1. For instance, a novel FMEA model in Li-En Wang's study combines the analytic network process (ANP) and the complex proportional assessment (COPRAS) to evaluate and rank failure modes in interval-valued intuitionistic fuzzy environments. This approach demonstrates its accuracy and flexibility in hospital service contexts by combining the advantages of interval-valued intuitionistic fuzzy sets with ANP and COPRAS for multi-criteria decision-making [38]. However, a few studies have employed MCDM approaches to assess fire safety.

**Table 1:** provides an overview of the studies that used MCDM approaches for risk assessments in healthcare facilities.

Tool type	Objective	Approach used	Study
FMEA, Fuzzy-AHP and Fuzzy-VIKOR	Occupational health and safety (OHS) risk assessment in hospital	Used Fuzzy-VIKOR to assess OHS risks in each hospital department and FAHP to calculate the relative weightings of the five risk factors.	[29]
Fuzzy-AHP and TOPSIS	an OHS policy to lower workplace accidents in hospital	Used TOPSIS to rank of the hazards in hospital and FAHP to determine the weights of the factors	[31]
Pythagorean fuzzy AHP (PF-AHP) and fuzzy TOPSIS	evaluation of the service quality in hospital	Used PF-AHP to determine the relative weightings of the 32 service quality evaluation criteria, and used Fuzzy TOPSIS to determine priorities of the healthcare centers	[42]
ANP^a^, reality-design gap evaluation and FIS	An anticipating risk assessment approach for an information system in hospitals	The gap between design and reality is used to estimate risk likelihood and determine the weights of the elements using ANP. Finally, the risk magnitude is determined using FIS.	[28]
FMEA, IVIF-COPRAS^b^, IVIF-ANP^c^	to assess and rank the risk of failure modes	To evaluate and prioritize failure mode risk by IVIF-COPRAS. Additionally, an IVIF-ANP is used to determine the risk factor weights.	[38]
HAZOP and Intuitionistic fuzzy sets	to identify the significant deviations in the oxygen supply system in hospitals	The conventional HAZOP approach categorized the risk levels into four groups based on crisp numbers. then, the output of the intuitionistic fuzzy set was divided into five categories.	[36]
Modified Delphi method, IVN-AHP and IVN-TOPSIS method	to assess fire risk after an earthquake in buildings.	The Modified Delphi approach is used to determine the criteria. First, the IVN-AHP approach determines the importance weights. The districts in Anatolian side of Istanbul are then ranked based on their post-earthquake fire hazards using the IVN-TOPSIS approach.	[39]
Fuzzy Delphi and AHP method	to estimate the degree of fire risk in hospitals	Fuzzy Delphi and AHP method were combined with Edinburgh point scheme as a scoring method to determine safety level	[43]
FMEA, IFM-BWM^d^ and IVIF-CODAS^e^	Fire risk assessment in hospital	The hospital wards are ranked using IVIF-CODAS after the weights of the factors are determined using IFM-BWM.	[44]
F-DEMATEL^f^ and Fuzzy Grey Relational Analysis (F-GRA)	to rank the risk levels of the emergency departments	risk criteria are assigned weights by fuzzy DEMATEL. F-GRA is used to obtain the rank of emergency departments according to the risk levels.	[35]
F-Delphi technique, IVN-AHP and FIS	Fire risk assessment in healthcare centers	determining main criteria and sub- criteria using systematic review and F-Delphi technique, weighting of the criteria using IVN-AHP method, and then estimating risk magnitude using FIS based on the PRL, RAL, and PL parameters.	Our study

a analytic network process.

b interval-valued intuitionistic fuzzy COmplex PRoportional ASsessment.

c interval-valued intuitionistic fuzzy ANP.

d Intuitionistic Fuzzy Multiplicative Best-Worst Method.

e Interval-Valued Intuitionistic Fuzzy Combinative Distance-based Assessment.

f Fuzzy Decision-Making Trial and Evaluation Laboratory.

Neutrosophic logic is a generalization of fuzzy logic. Moreover, Neutrosophic sets include uncertainty function in addition to the membership and non-membership functions. Therefore, it performs better than fuzzy sets to disambiguate experts' judgments and uncertainty in risk assessment. A two-level framework for post-earthquake fire risk assessment is presented by Gulum et al. utilizing literature review, Modified Delphi method, AHP technique, and NTOPSIS approach. The integrated methodology is put to the test using actual data to determine the riskiest districts in Istanbul, Turkey. For this purpose, the comprehensive technique aims to offer a reliable instrument for fire risk assessment and making decisions [39]. Another study used the Interval Valued Neutrosophic analytical hierarchy process (IVN-AHP) and fuzzy-Delphi method (F-Delphi) to identify and rank the occupational stressors among firefighters [40]. Omidvari et al. proposed a fire risk assessment in healthcare settings that combined FMEA with Multi-Criteria Decision Making (MCDM) techniques. The method involves determining weights using IFMBWM, and ranking different wards using IVIFCODAS [41]. Ultimately, The Interval Valued Neutrosophic AHP methodology serves as a powerful tool for capturing and managing the inherent vagueness, ambiguity, and indeterminacy associated with fire safety criteria and decision-making processes in healthcare environments. By allowing for interval-valued neutrosophic evaluations of criteria weights and pairwise comparisons, this methodological approach enables a more comprehensive and nuanced assessment of fire safety factors, such as evacuation procedures, fire detection systems, and emergency response protocols, within healthcare facilities.

Complementing the Interval Valued Neutrosophic-AHP, the incorporation of a Fuzzy Inference System enhances the predictive capabilities and modeling accuracy of the fire safety assessment framework enhances the predictive capabilities and modeling accuracy of the fire safety assessment framework by accommodating uncertainties, fuzzy inputs, and incomplete information in decision-making processes. Leveraging fuzzy logic principles and inference mechanisms, this system provides a flexible and adaptive approach to analyzing and synthesizing complex relationships between fire safety parameters, thereby enabling more robust and effective risk mitigation strategies in healthcare settings.

## 3 Methodology

This study has been carried out in three phases. First, the effective criteria on healthcare fire safety were discovered and categorized using the F-Delphi technique and literature review. In the second step, the weight of each factor was estimated using the IVN-AHP method. Three parameters (PRL, RAL and PL) as alternatives were calculated by IVN-AHP. Afterward, fire risk magnitude (FRM) and risk class were determined by fuzzy inference system (FIS). This section provides a detailed explanation of techniques that **were** employed in our proposed approach. Then, all of the steps in proposed approach are explained.

### 3.1 F-Delphi technique for determination candidate factors

**Fig. 1:**
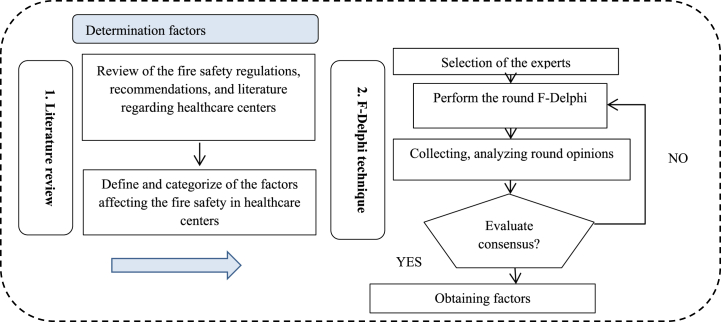
Steps of the determination candidate factors.

First, the effective criteria on healthcare fire safety were discovered and categorized using the F-Delphi technique and literature review, as shown in Fig. 1. A detailed review of healthcare-specific fire safety regulations, recommendations, and literature was conducted to identify the most appropriate criteria for fire risk in healthcare center. The presence of numerous parameters that impact the fire safety of healthcare buildings makes risk assessment process more difficult. On the other hand, the fire risk assessment process is a multidimensional and complex decision-making issue. To overcome this situation, following this literature review, the seven experts were consulted based on the F-Delphi technique to determine and categorize the most definitive and appropriate factors. In this case, an expert panel of seven members was invited with sufficient knowledge and expertise in the field of fire safety and fire risk assessment. Three managers responsible for firefighters, one for facilities, and three occupational health experts in the hospital were among the experts involved. Factors were extracted on a 5-point Likert scale in the questionnaire. That factor is eliminated if the computed average score below four has been determined. In the present study, F-Delphi consisted of four rounds.

### 3.2 Neutrosophic sets and preliminaries

Since Lotfi A. Zadeh introduced the fuzzy theory to the literature, many different approaches have been developed to handle ambiguous and uncertain knowledge. As a generalization of fuzzy sets that consider both memberships (truth) and non-memberships (falsity), Atanassov introduced intuitionistic fuzzy sets. To improve uncertainty handling, Smarandache created an advanced version of the intuitionistic fuzzy sets called neutrosophic logic [45].

Neutrosophic sets are distinguished by a truth-membership, an indeterminacy-membership, and a falsity-membership. However, fuzzy sets are not included indeterminacy-membership. Therefore, neutrosophic sets in comparison with fuzzy sets are among the more efficient and flexible sets to deal with inconsistency, uncertainty, and ambiguous information in decision-making processes.

Particularly, in decision-making issues where expert opinions are represented via linguistic terms, it allows experts more flexibility to share their opinions regarding the ambiguity and inconsistency of the information. Neutrosophic sets were applied in many studies to handle uncertain and ambiguous issues.

Fire risk assessment of the healthcare facilities investigated in this study involves numerous uncertainties regarding the nature of the issue and expert opinions. Interval valued neutrosophic sets are chosen to eliminate these uncertainties.

According to our study, the combination of AHP and FIS methods with neutrosophic sets and their utilization in the risk assessment issue has not been studied. As a result, a novel mathematical modeling for fire risk assessment was explored. Furthermore, this study presents the screening and selection of the most crucial factor for assessing the fire risk in healthcare facilities, which will bring innovation to the literature. In the following, basic concepts and definitions on an interval-valued Neutrosophic set are described.

Definition 1 $\tilde{\overset{\ldots }{A}}$ indicates a neutrosophic set E. Neutrosophic set is characterized by three memberships, including truth-membership function $T_{A}$, an indeterminacy-membership function $I_{A}$ and a falsity-membership function $F_{A}$.

$T_{A}\left(x\right)$, $I_{A}\left(x\right)$ and $F_{A}\left(x\right)$ are real standard subsets of $]0^{-},1^{+}[.\;A$ neutrosophic set $\tilde{\overset{\ldots }{A}}$ can be given by Eq. (1):

(1) $$\tilde{\overset{\ldots }{A}}=\{x,\left(T_{A}\left(x\right),I_{A}\left(x\right),F_{A}\left(x\right)\right)>:x\in E,(\{x,\left(T_{A}\left(x\right),I_{A}\left(x\right),F_{A}\left(x\right)\in ]0^{-},1^{+}[\right)\}$$

There is no restriction on the sum of $T_{A}\left(x\right)$, $I_{A}\left(x\right)$ and $F_{A}\left(x\right)$, therefore, $0^{-}\leq {\sup\;T}_{A}\left(x\right)+\sup\;I_{A}\left(x\right)+{\sup\;F}_{A}\left(x\right)\leq 3^{+}$.

Definition 2 Let X be a universe of discourse. An interval valued neutrosophic set $\tilde{\overset{\ldots }{N}}$ in E characterized by a truth-membership function $T_{N}\left(x\right)$, an indeterminacy-membership function $I_{N}\left(x\right)$ and a falsity-membership function $F_{N}\left(x\right)$ for each x∈X, where:

$$T_{N}\left(x\right)=\left[T_{N}^{L}\left(x\right),T_{N}^{U}\left(x\right)\right]\subseteq \left[0,1\right],I_{N}\left(x\right)=\left[I_{N}^{L}\left(x\right),I_{N}^{U}\left(x\right)\right]\subseteq \left[0,1\right],\text{and}\;F_{N}\left(x\right)=\left[F_{N}^{L}\left(x\right),F_{N}^{U}\left(x\right)\right]\subseteq \left[0,1\right]\text{.}$$

Thus, the interval-valued neutrosophic set $\tilde{\overset{\ldots }{N}}$ can be given by Eq. (2):

(2) $$\tilde{\overset{\ldots }{N}}=\{\left\langle \left[T_{N}^{L}\left(x\right),T_{N}^{U}\left(x\right)\right],\left[I_{N}^{L}\left(x\right),I_{N}^{U}\left(x\right)\right],\left[F_{N}^{L}\left(x\right),F_{N}^{U}\left(x\right)\right]\;\right\rangle |x\in X\}$$

Definition 3 Deneutrosophication: Bolturk and Kahraman proposed a deneutrosophication function of an interval-valued neutrosophic number is given in Eq. (3):

(3) $$D\left(x\right)=\left(\frac{\left(T_{N_{j}}^{L}\left(x\right)+T_{N_{j}}^{U}\left(x\right)\right)}{2}+\left(\left(I_{N_{j}}^{U}\right)\left(1-\frac{\left(I_{N_{j}}^{L}\left(x\right)+I_{N_{j}}^{U}\left(x\right)\right)}{2}\right)\right)-\left(\left(1-F_{N_{j}}^{U}\right)\times \frac{\left(F_{N_{j}}^{L}\left(x\right)+F_{N_{j}}^{U}\left(x\right)\right)}{2}\right)\right)$$

Where $\tilde{\overset{\ldots }{N_{j}}}=\left\langle \left[T_{N_{j}}^{L}\left(x\right),T_{N_{j}}^{U}\left(x\right)\right],\left[I_{N_{j}}^{L}\left(x\right),I_{N_{j}}^{U}\left(x\right)\right],\left[F_{N_{j}}^{L}\left(x\right),F_{N_{j}}^{U}\left(x\right)\right]\right\rangle$.

Definition 4 Let $\tilde{\overset{\ldots }{N_{1}}}=\left\langle \left[T_{N_{1}}^{L}\left(x\right),T_{N_{1}}^{U}\left(x\right)\right],\left[I_{N_{1}}^{L}\left(x\right),I_{N_{1}}^{U}\left(x\right)\right],\left[F_{N_{1}}^{L}\left(x\right),F_{N_{1}}^{U}\left(x\right)\right]\right\rangle$ and $\tilde{\overset{\ldots }{N_{2}}}=\left\langle \left[T_{N_{2}}^{L}\left(x\right),T_{N_{2}}^{U}\left(x\right)\right],\left[I_{N_{2}}^{L}\left(x\right),I_{N_{2}}^{U}\left(x\right)\right],\left[F_{N_{2}}^{L}\left(x\right),F_{N_{2}}^{U}\left(x\right)\right]\right\rangle$ be two interval-valued neutrosophic numbers. Their relations and arithmetic operations are given by Eqs. (4), (5), (6):

$$\tilde{\overset{\ldots }{{N_{1}}^{c}}}=\left\langle \left[T_{N_{1}}^{L}\left(x\right),T_{N_{1}}^{U}\left(x\right)\right],\left[{1-I}_{N_{1}}^{L}\left(x\right),{1-I}_{N_{1}}^{U}\left(x\right)\right],\left[F_{N_{1}}^{L}\left(x\right),F_{N_{1}}^{U}\left(x\right)\right]\right\rangle \text{.}$$

where $\tilde{\overset{\ldots }{{N_{1}}^{c}}}$ represents the complement o $\tilde{f}$ $\tilde{\overset{\ldots }{N_{1}}}$.

(4) $$\tilde{\overset{\ldots }{N_{1}}}⨁\tilde{\overset{\ldots }{N_{2}}}=\left[T_{N_{1}}^{L}+T_{N_{2}}^{L}{-T}_{N_{1}}^{L}T_{N_{2}}^{L},T_{N_{1}}^{U}+T_{N_{2}}^{U}{-T}_{N_{1}}^{U}T_{N_{2}}^{U}\right],\left[I_{N_{1}}^{L}I_{N_{2}}^{L},I_{N_{1}}^{U}I_{N_{2}}^{U}\right],\left[F_{N_{1}}^{L}F_{N_{2}}^{L},F_{N_{1}}^{U}F_{N_{2}}^{U}\right]$$

(5) $$\tilde{\overset{\ldots }{N_{1}}}⊗\tilde{\overset{\ldots }{N_{2}}}=\left[T_{N_{1}}^{L}T_{N_{2}}^{L},T_{N_{1}}^{U}T_{N_{2}}^{U}\right],\left[I_{N_{1}}^{L}+I_{N_{2}}^{L}{-I}_{N_{1}}^{L}I_{N_{2}}^{L},I_{N_{1}}^{U}+I_{N_{2}}^{U}{-I}_{N_{1}}^{U}I_{N_{2}}^{U}\right],\left[F_{N_{1}}^{L}+F_{N_{2}}^{L}{-F}_{N_{1}}^{L}F_{N_{2}}^{L},F_{N_{1}}^{U}+F_{N_{2}}^{U}{-F}_{N_{1}}^{U}F_{N_{2}}^{U}\right]$$

(6) $$\tilde{\overset{\ldots }{N_{1}}}⊖\tilde{\overset{\ldots }{N_{2}}}=\left[T_{N_{1}}^{L}-F_{N_{2}}^{U},T_{N_{1}}^{U}-F_{N_{2}}^{L}\right],\left[\max\left(I_{N_{1}}^{L}{,I}_{N_{2}}^{L}\right),\max\left(I_{N_{1}}^{U}{,I}_{N_{2}}^{U}\right)\right],\left[F_{N_{1}}^{L}-T_{N_{2}}^{U},F_{N_{1}}^{U}-F_{N_{2}}^{L}\right]$$

### 3.3 Interval-valued neutrosophic-AHP (IVN-AHP)

AHP is a multi-criteria decision-making approach that **was** introduced to the literature by Thomas Saaty (1970). It involves organizing a problem into hierarchies and evaluating the criteria in the hierarchy using pairwise comparisons [46].

Neutrosophic AHP can be used to handle uncertainty and inconsistency in data more effectively than classical AHP. In this study, the importance weights of each factor in the hierarchy affecting the fire risk in healthcare facilities are estimated using the interval-value neutrosophic-AHP approach. These importance weights are applied as the constant coefficients of each sub-factor in equations (10), (11), (12). The steps of IVN-AHP that are applied in fire risk problem are listed in section 3.5 (Steps 1–7).

### 3.4 Fuzzy inference system

A fuzzy inference system is a mapping input to output space using membership functions and fuzzy rules, providing a basis for finding patterns and decision-making. Mamdani- FIS, the first and most well-known fuzzy inference system, is employed in this work. The four steps of the fuzzy inference system (FIS) are fuzzification, knowledge base, fuzzy inference system, and defuzzification. These steps employed in this work are described in section 3.5 (Steps 8–12).

### 3.5 Steps of the proposed integrated method

**Fig. 2:**
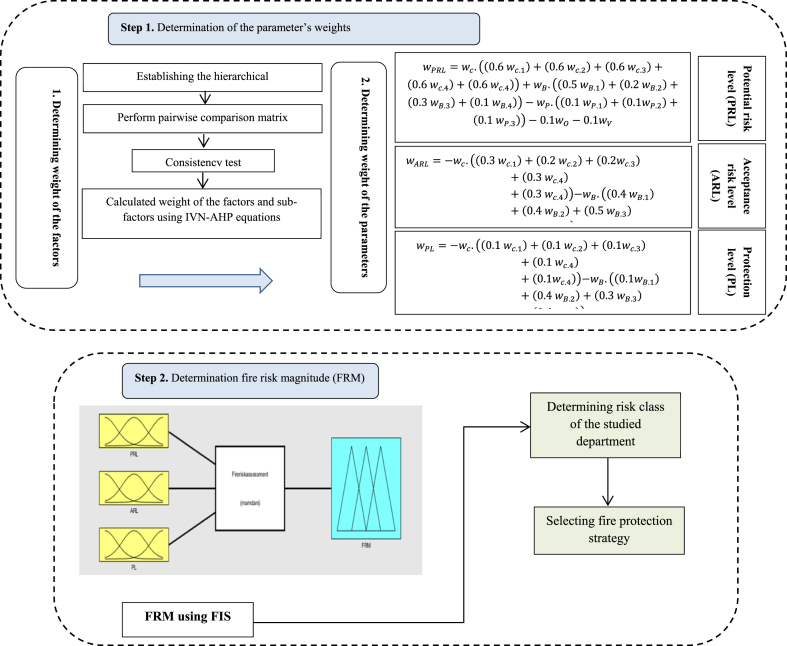
Steps of the proposed integrated method.

The proposed integrated approach is carried out in two phases, **as** shown in Fig. 2. The steps of the proposed integrated method for fire risk assessment are listed as follows.

**Fig. 3:**
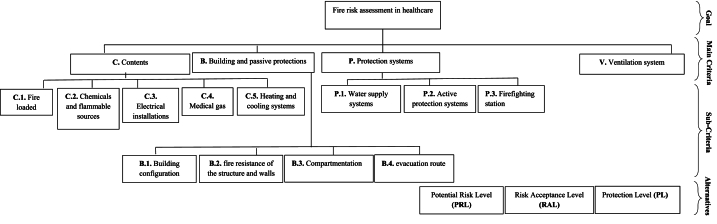
Analytical hierarchical process.

Step 1 The main factors and sub-factors for the fire risk problem were constructed in a hierarchical structure, as shown in Fig. 3. Three parameters including PRL, RAL and PL, were presented as alternatives.

Step 2 A pairwise comparison was constructed and then the linguistic terms as shown in Table 2 are identified by the expert group. The interval value neutrosophic numbers are substituted for the corresponding linguistic term in the pairwise comparison matrix. To ensure consistency, the pairwise comparison matrix is deneutrosophicated using Equation (3).

**Table 2:** Linguistic terms and interval valued neutrosophic (IVN).

Linguistic terms	$\left(T^{L},T^{U\;}\right)$	$\left(I^{L},I^{U}\;\right)$	($F^{L},F^{U}$)
Equal influential (EI)	(0.5, 0.5)	(0.5, 0.5)	(0.5, 0.5)
Weakly More influential (WMI)	(0.5, 0.6)	(0.35, 0.45)	0.4, 0.5)
Moderate influential (MI)	(0.55, 0.65)	(0.3, 0.4)	(0.35, 0.45)
Moderately More influential (MMI)	(0.6, 0.7)	(0.25, 0.35)	(0.3, 0.4)
Strong influential (SI)	(0.65, 0.75)	(0.2, 0.3)	(0.25, 0.35)
Strongly More influential (SMI)	(0.7, 0.8)	(0.15, 0.25)	(0.2, 0.3)
Very Strong influential (VSI)	(0.75, 0.85)	(0.1, 0.2)	(0.15, 0.25)
Very Strongly More influential (VSMI)	(0.8, 0.9)	(0.05, 0.1)	(0.1, 0.2)
Extreme influential (EXI)	(0.9, 0.95)	(0, 0.05)	(0.05, 0.15)
Extremely High influential (EHI)	(0.95, 1)	(0, 0)	(0, 0.1)
Absolutely More influential (AMI)	(1, 1)	(0, 0)	(0, 0)

If the deneutrosophicated pairwise comparison is consistent, it is possible to conclude that the neutrosophic pairwise comparison is consistent. The pairwise comparison matric ($\tilde{\overset{\ldots }{P}}$) of the factors are provided by equation (7).

(7) $$\tilde{\overset{\ldots }{P}}\begin{array}{cc} = & \;\begin{array}{c} \begin{array}{ccc} \begin{array}{cc} {\;C}_{1} & \;C_{2} \end{array} & \;\ldots & \;C_{n} \end{array}\\ \begin{array}{c} \begin{array}{c} C_{1}\\ C_{2} \end{array}\\ \begin{array}{c} \vdots \\ C_{n} \end{array} \end{array}\left[\begin{array}{ccc} \begin{array}{cc} {\tilde{\overset{\ldots }{A}}}_{11} & {\tilde{\tilde{\overset{\ldots }{A}}}}_{12}\\ {\tilde{\tilde{\overset{\ldots }{A}}}}_{21} & {\tilde{\tilde{\overset{\ldots }{A}}}}_{22} \end{array} & \begin{array}{c} \cdots \\ \cdots \end{array} & \begin{array}{c} {\tilde{\tilde{\overset{\ldots }{A}}}}_{1n}\\ {\tilde{\tilde{\overset{\ldots }{A}}}}_{2n} \end{array}\\ \begin{array}{cc} \vdots & \vdots \end{array} & \ddots & \vdots \\ \begin{array}{cc} {\tilde{\tilde{\overset{\ldots }{A}}}}_{m1} & {\tilde{\tilde{\overset{\ldots }{A}}}}_{m2} \end{array} & \cdots & {\tilde{\tilde{\overset{\ldots }{A}}}}_{mn} \end{array}\right] \end{array} \end{array}i=1,2,3,\ldots ,n\;;j=1,2,3,\ldots ,m$$

C is criteria or factor, in this study, ${\tilde{\tilde{\overset{\ldots }{A}}}}_{ij}=\left\langle \left(T_{ij}^{L},T_{ij}^{u}\right),\left(I_{ij}^{L}{,I}_{ij}^{u}\right),\left(F_{ij}^{L},F_{ij}^{u}\right)\right\rangle$ , m = n

Step 3 The normalized importance weight of the factor ($\tilde{\overset{\ldots }{N_{ij}}}$) is obtained using the following equation (8).

(8) $$\tilde{\overset{\ldots }{N_{ij}}}=\left\langle \left(\frac{T_{kj}^{L}}{\sum_{k=1}^{n}T_{kj}^{u}},\frac{T_{ij}^{u}}{\sum_{k=1}^{n}T_{kj}^{u}}\right),\left(\frac{I_{kj}^{L}}{\sum_{k=1}^{n}I_{kj}^{u}},\frac{I_{ij}^{u}}{\sum_{k=1}^{n}I_{kj}^{u}}\right),\left(\frac{F_{kj}^{L}}{\sum_{k=1}^{n}F_{kj}^{u}},\frac{F_{ij}^{u}}{\sum_{k=1}^{n}F_{kj}^{u}}\right)\right\rangle$$

Step 4 The arithmetic mean in each row is computed using Equation (9), which provides the neutrosophic important weight vector ($\tilde{\overset{\ldots }{W_{ij}}}$) for the factor.

(9) $$\tilde{\overset{\ldots }{W_{ij}}}=\left\langle \left(\frac{\sum_{k=1}^{n}\frac{T_{kj}^{L}}{\sum_{k=1}^{n}T_{kj}^{u}}}{n},\frac{\sum_{k=1}^{n}\frac{T_{ij}^{u}}{\sum_{k=1}^{n}T_{kj}^{u}}}{n}\right),\left(\frac{\sum_{k=1}^{n}\frac{I_{kj}^{L}}{\sum_{k=1}^{n}I_{kj}^{u}}}{n},\frac{\sum_{k=1}^{n}\frac{I_{ij}^{u}}{\sum_{k=1}^{n}I_{kj}^{u}}}{n}\right),\left(\frac{\sum_{k=1}^{n}\frac{F_{kj}^{L}}{\sum_{k=1}^{n}F_{kj}^{u}}}{n},\frac{\sum_{k=1}^{n}\frac{F_{ij}^{u}}{\sum_{k=1}^{n}F_{kj}^{u}}}{n}\right)\right\rangle$$

Step 5 the crisp weights of the factor are determined by deneutrosophication formula in Equation (3).

Step 6 The weights of each main factor, sub-factor, and alternatives were determined by repeating the aforementioned steps.

Step 7 The weight of the parameters (alternatives) is obtained by following equations (10), (11), (12). As explained in detail at section 2, 3, the constant coefficient in equations (10), (11), (12) represents importance weight of the alternatives with respect to those sub-factors.

(10) $$w_{PRL}=w_{c}.\left(\left(0.6\;w_{c.1}\right)+\left(0.6\;w_{c.2}\right)+\left(0.6\;w_{c.3}\right)+\left(0.6\;w_{c.4}\right)+\left(0.6\;w_{c.4}\right)\right)+w_{B}.\left(\left(0.5\;w_{B.1}\right)+\left(0.2\;w_{B.2}\right)+\left(0.3\;w_{B.3}\right)+\left(0.1\;w_{B.4}\right)\right)-w_{P}.\left(\left(0.1\;w_{P.1}\right)+\left(0.1w_{P.2}\right)+\left(0.1\;w_{P.3}\right)\right)-0.1w_{O}-0.1w_{V}$$

$w_{PRL}$ is weight of the potential risk level;

(11) $$w_{ARL}=-w_{c}.\left(\left(0.3\;w_{c.1}\right)+\left(0.2\;w_{c.2}\right)+\left(0.2w_{c.3}\right)+\left(0.3\;w_{c.4}\right)+\left(0.3\;w_{c.4}\right)\right){-w}_{B}.\left(\left(0.4\;w_{B.1}\right)+\left(0.4\;w_{B.2}\right)+\left(0.5\;w_{B.3}\right)+\left(0.3\;w_{B.4}\right)\right)+w_{P}.\left(\left(0.2w_{P.1}\right)+\left(0.2w_{P.2}\right)+\left(0.2\;w_{P.3}\right)\right)+0.5w_{O}+0.6w_{V}$$

$w_{ARL}$ is weight of the risk acceptance level;

(12) $$w_{PL}=-w_{c}.\left(\left(0.1\;w_{c.1}\right)+\left(0.1\;w_{c.2}\right)+\left(0.1w_{c.3}\right)+\left(0.1\;w_{c.4}\right)+\left(0.1w_{c.4}\right)\right){-w}_{B}.\left(\left(0.1w_{B.1}\right)+\left(0.4\;w_{B.2}\right)+\left(0.3\;w_{B.3}\right)+\left(0.6\;w_{B.4}\right)\right)+w_{P}.\left(\left(0.7w_{P.1}\right)+\left(0.7w_{P.2}\right)+\left(0.6w_{P.3}\right)\right)+0.4w_{O}+0.3w_{V}$$

**Fig. 4:**
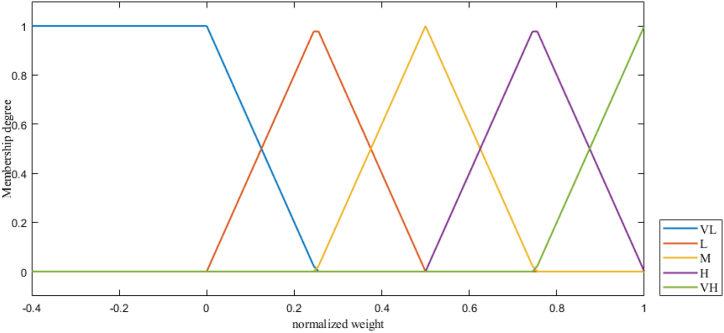
Membership functions of PRL, RAL and PL inputs. VL: Very Low, L: Low, M: Medium, H: High, and VH: Very High.

$w_{PL}$ is weight of the protection level;

Step 8 the crisp weight (obtained in step 7) is divided by the maximum weight of the three parameters to determine the normalized weight of the parameters. Using the obtained normalized weight, the degrees of membership for these three parameters are found using Fig. 4. This study employs a five-member linguistic term set, which includes very high (VH), high (H), medium (M), low (L), and very low (VL), to convert the normalized weight of all three parameters into a membership function. When each normalized weight of the parameters is a negative or less than zero, the membership degree of the very low (VL) linguistic term will be 1, as shown in Fig. 4.

**Fig. 5:**
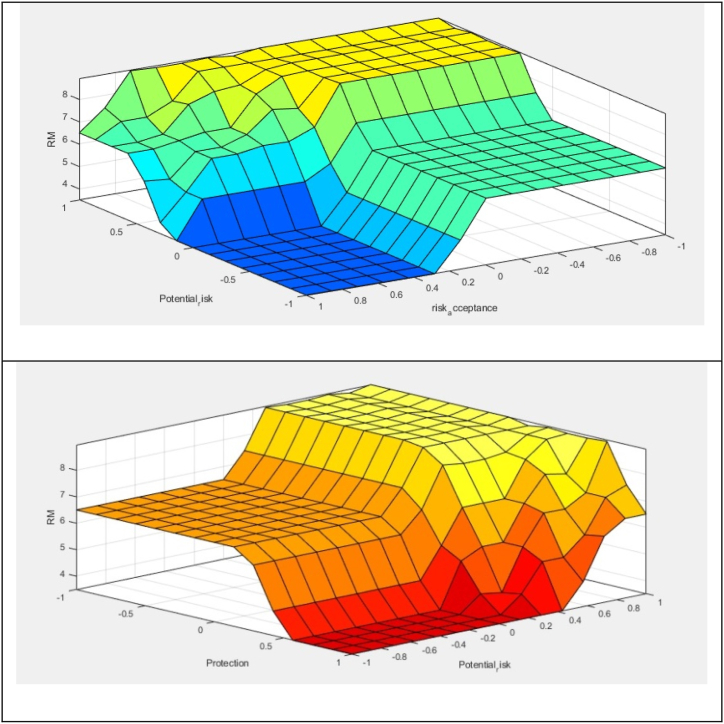
Rules of fuzzy inference system.

Step 9 This step provides the rule base when fuzzy "if-then" rules are defined. The following are the if-then rule structures used in this study. The rules of the fuzzy inference system in this study are presented in Fig. 5.

If $\mathrm{PR}\;is\;A_{i1\;},RA\;is\;A_{i2\;}\;and\;PL\;is\;A_{in\;}\;then\;RM\;is\;B_{i\;}$

Where $A_{in\;}$ are linguistic terms for membership function of input parameters (PR, RA and PL) and linguistic terms ($B_{i\;}$) for output (RM), respectively, in r_th_ rule.

Step 10 The fuzzy inference unit uses membership functions, fuzzy logic and if-then rules to perform the modeling process. The maximal composition used in this work is the most often used fuzzy inference unit approach. The following formula provides a mathematical description of this approach.

(13) $${\mu_{D}}_{r}\left(RM\right)=\max\left[\min\left[{\mu_{A}}_{r}\left(inpute\left(\mathrm{PR}\right)\right),{\mu_{B}}_{r}\left(input\left(RA\right)\right),\mu_{r}\left(input\left(PL\right)\right)\right]\right]\;r=1,2,\ldots$$

Where ${\mu_{D}}_{r}\left(\text{RM}\right)$ is membership of output (RM) for rth rule, ${\mu_{A}}_{r}$ $,{\mu_{B}}_{r}$, and ${\mu_{C}}_{r}$ are membership functions input “PRL”, “ARL”, and “PL”, respectively.

Step 11 In the Center of Area (COA) approach, one of the most popular defuzzification techniques, the fuzzy sets are converted to crisp values using equation (14).

(14) $$Z_{COA}^{*}=\frac{\int {z\mu }_{A}\left(z\right)dz}{\int \mu_{A}\left(z\right)dz}$$

**Fig. 6:**
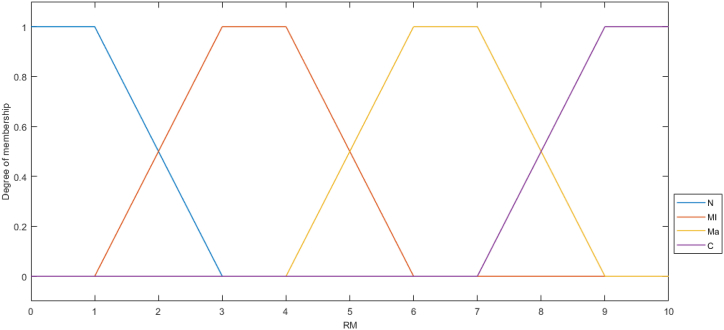
Membership functions of output. RM: risk magnitude, N: Negligible, Mi: Minor, Ma: Major, and C: Critical.

Step 12 Fig. 6 is used to determine risk class including Negligible, Minor, Major, and Critical by risk magnitude. Finally, based on the risk class of the department in healthcare centers, control strategy, including manual firefighting, automatic fire detection system, sprinkler protection, and preventive measures is suggested for Negligible, Minor, Major, and Critical fire risk class, respectively.

## 4 Results

### 4.1 Candidate factors obtained from literature review and F-Delphi technique

As mentioned, a detailed literature review was conducted to identify the most appropriate factor. As a result, 42 criteria regarding fire safety healthcare centers were discovered through the literature review.

Following this literature review, expert group was consulted based on the F-Delphi technique to determine and categorize the most definitive and appropriate factor, as explained in section 2.1. Finally, five main factors and eleven sub-factors for fire risk assessment are determined by F-Delphi technique. A brief explanation of each factor was presented in this section. Additionally, the results of the alternative weights for each sub-factor were provided. These weights are used as constant coefficients in equations 10–12.

#### 4.1.1 Contents

In proposed fire risk assessment method, building contents include oxygen, combustible materials, and ignition sources. In healthcare buildings, ignition sources and fire spread factors were considered sub-factor related to building contents. According to equations (10), (11), (12), these sub-factors have a positive impact on potential risk level, a negative impact on risk acceptance level, and almost no effect on the protection level.

Matrix of pairwise comparisons of the alternatives with respect to sub-factor was created. The experts answered the question, “How much effect does the each of the following sub-factor have on one parameter compared to another parameter?” the linguistic terms presented in Table 3, Table 4, Table 5, Table 6, Table 7, Table 8 was agreed upon by seven experts.

**Table 3:** Pairwise comparison matrix for parameters with respect to fire load.

parameters	PRL	RAL	PL	Weight
PRL	EI	EXI	AMI	0.57
RAL	${EXI}^{c}$	EI	SMI	0.28
PL	${\text{AMI}}^{c}$	${SMI}^{c}$	EI	0.13

**Table 4:** pairwise comparison matrix for parameters with respect to chemicals and flammable sources.

parameters	PRL	RAL	PL	weight
PRL	EI	EXI	AMI	0.57
RAL	${EXI}^{c}$	EI	SMI	0.28
PL	${\text{AMI}}^{c}$	${SMI}^{c}$	EI	0.13

**Table 5:** pairwise comparison matrix for parameters with respect to electrical installations.

parameters	PRL	RAL	PL	weight
PRL	EI	EHI	AMI	0.62
RAL	${EHI}^{c}$	EI	AMII	0.28
PL	${AMI}^{c}$	${AMI}^{c}$	EI	0.06

**Table 6:** pairwise comparison matrix for parameters with respect to medical gas.

parameters	PRL	RAL	PL	weight
PRL	EI	AMI	AMI	0.59
RAL	${AMI}^{c}$	EI	AMI	0.24
PL	${AMI}^{c}$	${AMI}^{c}$	EI	0.07

**Table 7:** pairwise comparison matrix for parameters with respect to heating and cooling systems.

parameters	PRL	RAL	PL	weight
PRL	EI	EHI	AMI	0.62
RAL	${EHI}^{c}$	EI	AMI	0.28
PL	${AMI}^{c}$	${AMI}^{c}$	EI	0.06

**Table 8:** pairwise comparison matrix for parameters with respect to building configuration.

parameters	PRL	RAL	PL	weight
PRL	EI	SI	AMI	0.50
RAL	${SI}^{c}$	EI	EHI	0.37
PL	${AMI}^{C}$	${EHI}^{c}$	EI	0.07

##### 4.1.1.1 Fire load

One of the most crucial factors in fire risk assessment is the fire load density of structural and contents building. The experts answered the question, “How much effect does the fire load have on one parameter compared to another parameter?” by creating a matrix of pairwise comparisons of the alternatives with respect to fire load according to Table 3. This allowed us to calculate the impact coefficient of the fire load on three alternatives (acceptable risk level, potential risk level, and protection level). For instance, the potential risk level relative to the acceptance risk level is extremely highly influenced by the immobile and mobile fire load. The coefficient of fire load on potential risk, risk acceptable level, and protection level is almost obtained to be 0.6, 0.3, and 0.1, respectively, as shown in Table 3.

##### 4.1.1.2 Chemicals and flammable sources

Chemicals are available in different departments of the hospital including labs, pharmacies and etc. The user of the proposed method should prepare material safety data sheet (MSDS) from the chemical supplier and find out the presence of flammable and combustible materials in the department. Table 4 displays the matrix of pairwise comparisons of alternatives with regard to Chemicals and flammable sources.

As shown in Table 4, the coefficient of Chemicals and flammable sources on possible risk, acceptable risk level and protection level were almost obtained as 0.6, 0.2 and 0.1, respectively.

##### 4.1.1.3 Electrical installations

Most departments in healthcare facilities have electrical equipment that may ignite fire. The use of combustible materials with electrical equipment is classified in the electrical codes because switchboards and electrical equipment generate sparks and have hot surfaces. In these sub-factors, the user must be considering short circuits, damaged cables, transformers, storage, and electrical installations, as well as regular inspections and regulation compliance. Table 5 displays the matrix of pairwise comparisons of alternatives with regard to electrical installations. Potential risk (effect coefficient = 0.6) and Risk acceptance (effect coefficient = 0.3) have identical weights to all the sub-factor of the contents.

##### 4.1.1.4 Medical gases

Flammable medical gas (Acetylene, Butane, Ammonia, Ethane, and Propane, and etc.) and non-flammable medical gas (compressed oxygen, helium, nitrogen, and etc.) are used in ICU, NICU, operating room and most inpatient departments. Their use can exacerbate the risk of fire in medical centers. Numerous studies have revealed that hospital oxygen cylinders led to catastrophic fires. In fact, it is an important factor in the fire spread. Therefore, it is necessary to ensure that the storage of medical gas cylinders complies with regulations in the first risk assessment of healthcare buildings.

Table 6 shows the matrix of paired comparisons based on expert judgment for alternatives with respect to medical gas. The importance weight of medical gas on risk acceptance level, potential risk level, and protection level were 0.6, 0.2, and 0.1, respectively, as Table 6 illustrates.

##### 4.1.1.5 Heating and cooling systems

Heating systems are one well-known fire sources. There are no special risks associated with systems that use hot water or steam. However, systems which utilize combustible thermal fluids instead of water are potentially hazardous. Furthermore, dust and oil may accumulate in air conditioning systems, and they can transfer smoke and burning particles from one place to another. The electrical heating system establishes a potential fire risk when the sensors in these systems fail. The greatest fire risk is associated with heating systems that include open flames or hot radiating surfaces.

The user can refer to the European ATEX 137 directive or NFPA 70 of the National Electrical Code, which classify hazardous zones, to assist them make an informed decision-making. The matrix of pairwise comparisons for parameters regard to Heating and cooling systems is displayed in Table 7.

#### 4.1.2 Building and passive protections

Building elements and passive protection systems in order to reduce the heat transmission and fire spread are critical to fire safety. Building configuration, fire resistance of the structure and walls, Compartmentation and evacuation route were chosen by expert group. In the following, each of the sub-factors was explained in detail.

As in the previous section, a matrix of pairwise comparisons of alternatives with respect to every sub-criterion was formed.

As (10), (11), (12) reveals, these sub-factors have a negative effect on risk acceptance and a positive effect on potential risk. Therefore, these sub-factors with higher weight have a stronger effect on fire risk. As a result, potential risk rises and risk acceptance falls following more weight of these sub-factor.

The user answers the question which sub-factor has a greater impact on the fire risk than the other sub-factor. The user should consider the description of the following sub-factor. Afterward, for example, if department in healthcare buildings, the evacuation route is inappropriate than the compartmentation. The user of the pairwise comparison chooses the more effect of the evacuation route than the compartmentation, but which linguistic terms according to Table 2 to choose depends on the degree of its impact on the fire risk.

##### 4.1.2.1 Building configuration

This sub-criterion should be investigated adequate open space, building height, and building location relative to other nearby buildings, that are crucial significance in the fire safety assessment in healthcare buildings. Sufficient open space should be available both inside and outside healthcare buildings to allow for the unimpeded movement of patients and emergency/firefighting vehicles in emergency situations. Reference height for aerial ladders is 25 m, in most countries. Therefore, higher floor become more difficult to access, which raises the fire risk. Building configuration impacts potential risk, risk acceptance level, and protection by 0.5, 0.4, and 0.1, as Table 8 indicates.

##### 4.1.2.2 Fire resistance

This sub-factor investigates the building's ability to resist the fire destructive effects. It is crucial to consider the fire resistance of the outside and inside walls, ceiling, and roof in addition to the building's structure. The ratings of the elements will be expressed in minutes as defined by tests based on ISO R 834. As shown in Table 9, the effect coefficient on potential risk level, risk acceptance level, and protection level was found to be 0.2, 0.4, and 0.4, respectively.

**Table 9:** pairwise comparison matrix for parameters with respect to fire resistance.

parameters	PRL	RAL	PL	weight
PRL	EI	${EHI}^{c}$	${SMI}^{c}$	0.16
RAL	EHI	EI	${MI}^{c}$	0.39
PL	SMI	MI	EI	0.42

##### 4.1.2.3 Compartmentation

Compartmentation provides barriers to the fire and lowers the number of occupants who need to be evacuated. Healthcare buildings can be designed so that there are two compartments on each floor, connected by fire door-protected apertures. When the Compartmentation of the building is deemed unacceptable, its weight will be high in pairwise comparisons regarding sub-factor of building and passive protections. As shown in Table 10, the effect coefficient on potential risk level, risk acceptance level, and protection level was found to be 0.3, 0.5, and 0.3, respectively.

**Table 10:** pairwise comparison matrix for parameters with respect to compartmentation.

parameters	PRL	RAL	PL	weight
PRL	EI	${EXI}^{c}$	${VSMI}^{C}$	0.30
RAL	EXI	EI	VSI	0.48
PL	VSMI	${VSI}^{c}$	EI	0.32

##### 4.1.2.4 Evacuation route

It is obvious that the exits should be at least as wide as the beds in the healthcare centers because its occupants require assistance to evacuate. On the other hand, in the fire safety analysis of buildings, the required safe egress time (RSET) should be less than available safe egress time (ASET) from the department. The number of exits with the required width must be sufficient in relation to the occupancy density in accordance to regulation of the healthcare building. The impact of evacuation route on risk acceptance level, potential risk level and protection are 0.1, 0.3 and 0.6 respectively, as shown in Table 11.

**Table 11:** pairwise comparison matrix for parameters with respect to evacuation route.

parameters	PRL	RAL	PL	weight
PRL	EI	${AMI}^{c}$	${AMI}^{c}$	0.06
RAL	AMI	EI	${VSMI}^{c}$	0.34
PL	AMI	VSMI	EI	0.57

#### 4.1.3 Protection systems

One of the most crucial factors in determining a building's level of fire safety is the existence of fire protection systems. The following three sub-factor were taken into consideration in the proposed method: 1. water supply systems; 2. active fire protection systems (fire extinguishing systems, portable fire extinguishers, variable detectors that trigger an alarm or automatic sprinkler, etc.); and 3. fire station. Table 12, Table 13, Table 14 displays the matrix of pairwise comparisons of alternatives with regard to these sub-factors.

**Table 12:** pairwise comparison matrix for parameters with respect to water supply systems.

parameters	PRL	RAL	PL	weight
PRL	EI	${AMI}^{c}$	${AMI}^{c}$	0.07
RAL	AMI	EI	${AMI}^{c}$	0.24
PL	AMI	AMI	EI	0.69

**Table 13:** pairwise comparison matrix for parameters with respect to active protection systems.

parameters	PRL	RAL	PL	Weight
PRL	EI	${AMI}^{c}$	${AMI}^{c}$	0.07
RAL	AMI	EI	${AMI}^{c}$	0.24
PL	AMI	AMI	EI	0.69

**Table 14:** pairwise comparison matrix for parameters with respect to firefighting station.

parameters	PRL	RAL	PL	Weight
PRL	EI	${EHI}^{c}$	${AMI}^{c}$	0.05
RAL	EHI	EI	${EXI}^{c}$	0.30
PL	AMI	EXI	EI	0.59

In pairwise comparison, the user must assess sub-factor of fire protection systems based on present regulations and guidelines. For example, in the water supply systems, the water source that is continuously needed to extinguish the fire should be checked. In most guidelines, the storage volume should be able to supply more than 2 h with enough pressure. Since the purpose of this study is not to provide guidelines or codes regarding parameters, we declined to involve more explanations.

#### 4.1.4 Organization elements

The user of the proposed approach ought to check whether the department has managed to put the organizational components such as training, disaster and emergency preparedness, fire exit drills and orders.

The high weight of this factor in the paired comparisons of the main factor indicates that its impact is unfavorable than another factor. Therefore, the organizational elements need to be improved.

The weight of the potential risk, risk acceptance, and protection influenced by 0.1, 0.5, and 0.4 wt of this criterion is according to (10), (11), (12), which is obtained based on pairwise comparisons of alternatives with respect to organization elements that is shown in Table 15.

**Table 15:** pairwise comparison matrix for parameters with respect to organization.

parameters	PRL	RAL	PL	Weight
PRL	EI	${AMI}^{c}$	${AMI}^{c}$	0.07
RAL	AMI	EI	SI	0.52
PL	AMI	${SI}^{c}$	EI	0.40

#### 4.1.5 Ventilation and smoke venting system

Adequate venting capacity for a developing fire is essential for smoke venting. Types of Smoke Management Systems including mechanical smoke exhaust and natural smoke ventilation should be considered. In pairwise comparisons of alternatives with respect to smoke venting systems, the effect coefficient on potential risk, risk acceptance, and protection was found to be 0.1, 0.6, and 0.3, respectively, as shown in Table 16.

**Table 16:** pairwise comparison matrix for parameters with respect to smoke venting system.

parameters	PRL	RAL	PL	Weight
PRL	EI	${AMI}^{c}$	${AMI}^{c}$	0.06
RAL	AMI	EI	EXI	0.59
PL	AMI	${EXI}^{c}$	EI	0.31

### 4.2 Application of the proposed approach in a public hospital

The proposed method was used to assess the fire risk in each department of a hospital in Iran. Owing to space constraints, only the steps required for the fire risk assessment of operating rooms of the hospital were provided in detail. This operating room department consists of seven operating rooms, each with a scrub unit and a warehouse. In addition, there are a recovery room, a CSR room, a doctor's office, a classroom, a reception area, and a dressing room. The proposed method can be used for each ward individually. It is preferable to assess each room. However, the fire risk assessment using proposed method can be used for an entire department. In this case, the worst-case scenario or the average of the department's characteristics should be considered. The operating room department characteristics are introduced to Table 17. The fire risk assessment steps of the proposed method are listed below.

Step 1 the expert must be determined which factor is more effective than other factor with regard to the fire risk target based on the IVN scales of the linguistic terms (as shown in Table 2). For example, in considering this present condition, medical gas is very strongly more influential (VSMI) than the heating and cooling system. For the operating room department, the consensus of the opinion experts on the pairwise comparison matrices for the main factor and their sub factor is given in Table 18, Table 19, Table 20, Table 21. On the other hand, the pairwise comparisons of the fire protection systems are based on their effect on fire suppression. For example, water supply system has a more moderate influential (MI) on the fire risk than the active protection systems since there are actually insufficient active protection devices in this operating room, such as fire detectors, extinguishers, and sprinklers.

**Table 18:** Pairwise comparison matrix for main criteria by linguistic terms based on expert opinion.

	C	B	P	O	V	
C	EI	VSMI	SMI	EHI	EXI	0.31
B	${VSMI}^{c}$	EI	MI	VSI	VSI	0.21
P	${SMI}^{c}$	${MI}^{c}$	EI	SMI	SMI	0.21
O	${EHI}^{c}$	${VSI}^{c}$	${SMI}^{c}$	EI	${MMI}^{c}$	0.11
V	${EXI}^{c}$	${VSI}^{c}$	${SMI}^{c}$	MMI	EI	0.13

**Table 19:** Pairwise comparison matrix for sub-criteria of the contents by linguistic terms.

	C.1	C.2	C.3	C.4	C.5	
C.1	EI	${EHI}^{c}$	${SMI}^{c}$	${EXI}^{c}$	${SI}^{c}$	0.10
C.2	EHI	EI	${MMI}^{c}$	${SI}^{c}$	VSMI	0.21
C.3	SMI	MMI	EI	${VSI}^{c}$	SMI	0.23
C.4	EXI	SI	VSI	EI	${SMI}^{c}$	0.25
C.5	SI	${VSMI}^{c}$	${SMI}^{c}$	SMI	EI	0.20

**Table 20:** Pairwise comparison matrix for sub-criteria of the building and passive protections by linguistic terms.

	B.1	B.2	B.3	B.4	
B.1	EI	MMI	VSMI	${EHI}^{c}$	0.21
B.2	${MMI}^{c}$	EI	MI	${EXI}^{c}$	0.20
B.3	${VSMI}^{c}$	${MI}^{c}$	EI	${AMI}^{c}$	0.12
B.4	EHI	EXI	AMI	EI	0.44
B.5	B.1	B.2	B.3	B.4

**Table 21:** Pairwise comparison matrix for fire protection systems by linguistic terms.

	P.1	P.2	P.3	
P.1	EI	MI	${SI}^{c}$	0.31
P.2	${MI}^{c}$	EI	WMI	0.33
P.3	SI	${WMI}^{c}$	EI	0.36

Step2 In accordance with Table 2, the linguistic terms are converted the interval-valued neutrosophic values. The pairwise comparison matrices incorporating interval-valued neutrosophic values for the main factor are presented in Table 22. Following that, as stated in section 2.2, the pairwise comparison matrix is checked for consistency, and it is discovered to be consistent.

**Table 22:** Pairwise comparison matrix by interval-valued neutrosophic values for the main criteria.

	C						B						P					
	$T^{L}$	$T^{U\;}$	$I^{L}$	$I^{U}$	$F^{L}$	$F^{U}$	$T^{L}$	$T^{U\;}$	$I^{L}$	$I^{U}$	$F^{L}$	$F^{U}$	$T^{L}$	$T^{U\;}$	$I^{L}$	$I^{U}$	$F^{L}$	$F^{U}$
**C**	0.5	0.5	0.5	0.5	0.5	0.5	0.8	0.9	0.05	0.1	0.1	0.2	0.7	0.8	0.15	0.25	0.2	0.3
**B**	0.1	0.2	0.9	0.95	0.8	0.9	0.5	0.5	0.5	0.5	0.5	0.5	0.55	0.65	0.3	0.4	0.35	0.45
**P**	0.2	0.3	0.75	0.85	0.7	0.8	0.35	0.45	0.6	0.7	0.55	0.65	0.5	0.5	0.5	0.5	0.5	0.5
**O**	0	0.1	1	1	0.95	1	0.15	0.25	0.8	0.9	0.75	0.85	0.2	0.3	0.75	0.85	0.7	0.8
**V**	0.05	0.15	0.95	1	0.9	0.95	0.15	0.25	0.8	0.9	0.75	0.85	0.2	0.3	0.75	0.85	0.7	0.8

	**O**						**V**											
	$T^{L}$	$T^{U\;}$	$I^{L}$	$I^{U}$	$F^{L}$	$F^{U}$	$T^{L}$	$T^{U\;}$	$I^{L}$	$I^{U}$	$F^{L}$	$F^{U}$						

**C**	0.95	1	0	0	0	0	0.9	0.95	0	0.05	0.05	0.15						
**B**	0.75	0.85	0.1	0.2	0.15	0.25	0.75	0.85	0.1	0.2	0.15	0.25						
**P**	0.7	0.8	0.15	0.25	0.2	0.3	0.7	0.8	0.15	0.25	0.2	0.3						
**O**	0.5	0.5	0.5	0.5	0.5	0.5	0.3	0.4	0.65	0.75	0.6	0.7						
**V**	0.6	0.7	0.25	0.35	0.3	0.4	0.5	0.5	0.5	0.5	0.5	0.5

Step 3 The importance weights of the factor are normalized by equation (8). The normalized pairwise comparison matrix for main factor is given in Table 23. Following that, Table 24 displays the calculated importance weights for each of the main factor.

**Table 23:** Pairwise comparison matrix by normalized- IVN values for the main criteria.

	C						B						P					
	$T^{L}$	$T^{U\;}$	$I^{L}$	$I^{U}$	$F^{L}$	$F^{U}$	$T^{L}$	$T^{U\;}$	$I^{L}$	$I^{U}$	$F^{L}$	$F^{U}$	$T^{L}$	$T^{U\;}$	$I^{L}$	$I^{U}$	$F^{L}$	$F^{U}$
**C**	0.40	0.40	0.12	0.12	0.12	0.12	0.34	0.38	0.02	0.03	0.03	0.07	0.27	0.31	0.05	0.09	0.07	0.11
**B**	0.08	0.16	0.21	0.22	0.19	0.22	0.21	0.21	0.16	0.16	0.16	0.16	0.22	0.25	0.11	0.14	0.12	0.16
**P**	0.16	0.24	0.17	0.20	0.17	0.19	0.15	0.19	0.19	0.23	0.18	0.21	0.20	0.20	0.18	0.18	0.18	0.18
**O**	0.00	0.08	0.23	0.23	0.23	0.24	0.06	0.11	0.26	0.29	0.25	0.28	0.08	0.12	0.26	0.30	0.25	0.28
**V**	0.04	0.12	0.22	0.23	0.22	0.23	0.06	0.11	0.26	0.29	0.25	0.28	0.08	0.12	0.26	0.30	0.25	0.28

	**O**						**V**											
	$T^{L}$	$T^{U\;}$	$I^{L}$	$I^{U}$	$F^{L}$	$F^{U}$	$T^{L}$	$T^{U\;}$	$I^{L}$	$I^{U}$	$F^{L}$	$F^{U}$						

**C**	0.25	0.26	0.00	0.00	0.00	0.00	0.26	0.27	0.00	0.03	0.03	0.08						
**B**	0.19	0.22	0.08	0.15	0.10	0.17	0.21	0.24	0.06	0.11	0.08	0.13						
**P**	0.18	0.21	0.12	0.19	0.14	0.21	0.20	0.23	0.09	0.14	0.11	0.16						
**O**	0.13	0.13	0.38	0.38	0.34	0.34	0.09	0.11	0.37	0.43	0.32	0.37						
**V**	0.16	0.18	0.19	0.27	0.21	0.28	0.14	0.14	0.29	0.29	0.26	0.26

**Table 24:** IVN importance weight and crisp weight of the main factor.

	$T^{L}$	$T^{U\;}$	$I^{L}$	$I^{U}$	$F^{L}$	$F^{U}$	crisp weight
**C**	0.3038	0.3256	0.0370	0.0530	0.0500	0.0741	0.31
**B**	0.1835	0.2183	0.1220	0.1581	0.1324	0.1685	0.21
**P**	0.1774	0.2128	0.1489	0.1868	0.1535	0.1892	0.21
**O**	0.0716	0.1096	0.3020	0.3269	0.2762	0.3027	0.11
**V**	0.0962	0.1337	0.2440	0.2752	0.2357	0.2655	0.13

Step 4 The deneutrosophication formula in Equation (2) is used to calculate the crisp weights after the neutrosophic importance weights of the factor have been determined. Table 24 provides the crisp weight for each main factor. These steps are repeated for each sub-factor. Importance weight of the whole factor for operating room are obtained and given in Table 25.

**Table 25:** Importance weight of main criteria and sub-criteria.

main criteria and sub-criteria	Importance weight for operating room	parameters		
		PRL	RAL	PL
Contents	0.31			
C.1. Fire loaded	0.10	0.6	0.3	0.1
C.2. Chemicals and flammable sources	0.23	0.6	0.2	0.1
C.3. Electrical installations	0.25	0.6	0.3	0.1
C.4. Medical gas	0.21	0.6	0.2	0.1
C.5. Heating and cooling systems	0.20	0.6	0.3	0.1
Building and passive protections	0.21			
B.1. Building configuration	0.21	0.5	0.4	0.1
B.2. fire resistance	0.20	0.2	0.4	0.4
B.3. Compartmentation	0.12	0.3	0.5	0.3
B.4. evacuation route	0.44	0.1	0.3	0.6
Protection systems	0.21			
P.1. Water supply systems	0.31	0.1	0.2	0.7
P.2. Active protection systems	0.33	0.1	0.2	0.7
P.3. Firefighting station	0.3	0.1	0.2	0.6
Organization elements	0.11	0.1	0.5	0.4
Ventilation system	0.13	0.1	0.6	0.3

As shown in Table 25, the importance weight of the building contents is higher than that of other main factors. Electrical equipment, chemicals, and medical gases have importance weights of 0.25, 0.23, and 0.21 among its sub-factor, respectively, which are greater than those of other sub-factors. Electrical equipment has the highest weight of fire risk factor (0.25) among the content factor in the operating room department. It is essential that measures be taken to improve the parameter with the highest importance weight.

In addition, the evacuation route has the greatest fire risk rating (0.44) among the building sub-factor.

Second floor in this hospital has just one rout, it is inadequate based on the volume of the occupants, and thus action and measures need to be taken. Other risky sub-factor in the operating room is the non-existence of active protection systems (rate: 0.33) and the deficiency water supply system (0.31).

Step 5 PRL, ARL and PL were estimated as AHP alternatives using (10), (11), (12). For the operating room, the importance weight of potential risk, acceptance risk level and fire protection level were obtained as 0.19, 0.06 and 0.06, respectively. Since the potential risk exceeds the accepted risk, the fire risk is considered unacceptable.

**Fig. 7:**
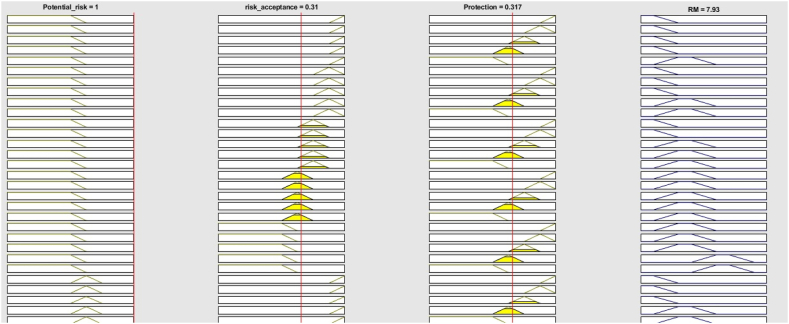
Rule of the FIS.

Step 6 As explained in section 3.2, the normalized weight of the parameters was obtained. The PRL, ARL, and PL have normalized weights of 1, 0.311, and 0.317, respectively. The risk magnitude for the operating room was calculated using steps 1–3 in section 3.2, and the result is 7.93, as Fig. 7 demonstrates. The risk class for this department was determined to be major, with a rate of 54 %, and critical, with a rate of 46 %, indicating that preventive measures are preferable for this class.

All the steps mentioned above were done for all departments in the hospital. As can be seen in Table 26, the departments of utilities and kitchen were determined above fifty percent in critical class. Also, the operating room (46.5 %), laundry (45 %), NICU 1 (45 %) and 2 (33.5 %) were determined to be critical. However, more than 50 % of the departments are in the major risk class. The utilities department in this hospital has the highest fire risk. Therefore, the top priority in fire risk management should be given to this department. According to the obtained results, none of the departments are classified in a class with a negligible fire risk. As a result, the use of automatic protection systems and sufficient water supply systems should be implemented for all departments.

**Table 26:** Results of the fire risk assessment in hospital.

Department	PRL	ARL	PL	RM	N	Mi	Ma	C
Room operation	0.19001	0.05918	0.06027	7.93	0	0	0.5350	0.4650
infectious	0.0723	0.217	0.265	4.66	0	0.6700	0.3300	0
Pathology	0.135	0.125	0.174	5.86	0	0.0700	0.9300	0
waste disposal	0.241	0.0482	0.1342	7.57	0	0	0.7150	0.2850
endoscopy	0.1945	0.0856	0.1582	6.46	0	0	1	0
Internal medicine specialist	0.0974	0.279	0.192	4.71	0	0.6450	0.3550	0
General Internal medicine	0.0987	0.3674	0.186	4.8	0	0.6000	0.4000	0
Laboratory	0.191	0.0147	0.106	7.38	0	0	0.8100	0.1900
pharmacy	0.1872	0.228	0.0835	5.73	0	0.1350	0.8650	0
kitchen	0.317	−0.0045	0.0562	8.85	0	0	0.0750	0.9250
utilities	0.2975	−0.0142	0.0645	8.86	0	0	0.0700	0.9300
Laundry	0.1965	−0.103	0.0915	7.9	0	0	0.5500	0.4500
Radiology - CT scan	0.064	0.108	0.156	5.54	0	0.2300	0.7700	0
Property warehouse	0.0764	0.259	0.245	4.49	0	0.7550	0.2450	0
post NICU 1	0.2645	0.0764	0.154	7.67	0	0	0.6650	0.3350
ICU1	0.198	0.0972	0.178	6.61	0	0	1	0
ICU 2	0.2093	0.0514	0.0352	7.15	0	0	0.9250	0.0750
maternity hospital	0.085	0.129	0.124	5.5	0	0.2500	0.7500	0
post-NICU 2	0.295	0.0701	0.106	7.65	0	0	0.6750	0.3250
poisoned department	0.0765	0.142	0.164	5.31	0	0.3450	0.6550	0
administrative department	0.0682	0.154	0.156	5.14	0	0.4300	0.5700	0
Library	0.0865	0.157	0.164	5.24	0	0.3800	0.6200	0
Pediatrics	0.138	0.241	0.178	5.18	0	0.4100	0.5900	0
special surgery inpatient	0.0954	0.164	0.181	5.26	0	0.3700	0.6300	0
NICU	0.243	0.097	0.105	7.36	0	0	0.8200	0.1800
angiography	0.0946	0.137	0.165	5.47	0	0.2650	0.7350	0
education	0.0913	0.164	0.189	5.22	0	0.3900	0.6100	0
LDR	0.0925	0.139	0.167	5.44	0	0.2800	0.7200	0
emergency room	0.0722	0.289	0.241	4.44	0	0.7800	0.2200	0
MRI	0.052	0.306	0.253	4.22	0	0.8900	1.1100	0

**Table 17:** Summary of the operating room features.

**contents of department (flammable and combustible sources)**	fire load	The movable fire load is chosen to be 600 MJ due to an ordinary fire hazard with low fire load according to standard (OH1/NFPA: OH Gp1). The immovable fire load is determined to be 100 MJ due to non-combustible construction with a maximum 10 % allowance for combustible building parts.
	medical gases	Medical gas cylinders including oxygen, nitrous oxide, medical air, and helium were available. The arrangement and storage of the cylinders near to the electrical equipment in operating room did not follow the regulations.
	Chemicals and flammable sources	Alcohol, Formalin, Sodium hypochlorite, Glutaraldehyde, formaldehyde and ethylene oxide.
	Electrical installations	In the operating room, there was numerous electrical equipment including a ventilator, anesthetic machine, cryosurgery, multi-channel electrocardiograph, medical monitor, etc. There are low-quality electrical installations and cables. Regular inspections of electrical appliances are carried out by the installation team.
	Heating and cooling systems	Heat transfer was through water and steam. The condition of the heating and cooling system was deemed appropriate.
**Building and passive protections (B)**	B.1. Building configuration	Height of the department: 3 m, height of the ground level: 6 m, and second floor; Area in total: 467 m
	B.2. resistance of the structure and walls	In the worst scenario, the building structure is fire resistant for 40 min. The resistance of internal materials is 35 min.
	B.3. compartmentation	Sub-segregation within fire areas with a maximum area of 1000 m^2^
	B.4. evacuation route	There is only one route on the second floor to the ground floor exit, and evacuation routes are not clearly marked. The shelter zone ends at the balcony. There is an enclosed internal staircase.
**fire protection systems**	1. Water supply systems	The operating room department has two fireboxes; however they do not cover every ward. Water supply is available for emergency needs, such as extinguishing fires, and is filled automatically.
	P.2. Active protection systems	A fire alarm is activated. There is no automatic fire detector. Sprinklers and an automated fire extinguishing system were unavailable.
	P.4. Firefighting station	Firefighters can be at the department in less than 5 min. This department has a team that has received firefighting training. It is difficult for firefighters to access the operating room.
**Organization elements (O)**	Disaster preparedness and fire exit drills and orders were not available. Occupants received firefighting training.	
**Ventilation and exhaust smoke system (V)**	The ventilation capacity is insufficient.	

### 4.3 Validation of the proposed method

Among the fire risk assessment methods, the Fire Risk Assessment Method for Engineers (FRAME) is the result of a 30-year search for a practical tool in fire safety assessment in buildings [47].

Numerous studies have been investigated the fire risk of the hospitals using this method [[48], [49], [50], [51], [52]]. To validate the proposed integrated method, we compared the fire risk magnitude for occupants and building obtained from FRAME method with the risk magnitude obtained from proposed method.

As shown in Table 27, there is no significant difference in the ranking of the studied hospital departments in both methods.

**Table 27:** Fire risk magnitude (FRM) in different department using FRAME and proposed methods.

department	proposed method						FRAME method			
	FRM	N	Mi	Ma	C	Rank	FRM for occupants	Rank	FRM for buildings	Rank
utilities	8.86	0	0	0.0700	0.9300	1	3.199	1	1.612	1
kitchen	8.85	0	0	0.0750	0.9250	2	2.509	2	1.207	3
Room operation	7.93	0	0	0.5350	0.4650	3	2.413	3	1.223	2
Laundry	7.9	0	0	0.5500	0.4500	4	1.959	4	1.158	4
post NICU 1	7.67	0	0	0.6650	0.3350	5	1.764	6	1.097	9
post-NICU 2	7.65	0	0	0.6750	0.3250	6	1.541	7	1.158	5
waste disposal	7.57	0	0	0.7150	0.2850	8	1.764	5	0.723	10
Laboratory	7.38	0	0	0.8100	0.1900	7	1.524	9	0.527	17
NICU	7.36	0	0	0.8200	0.1800	9	1.541	8	1.106	6
ICU 2	7.15	0	0	0.9250	0.0750	10	1.521	11	0.656	13
ICU 1	6.61	0	0	1	0	11	1.521	10	0.460	21
endoscopy	6.46	0	0	1	0	12	1.508	13	0.249	28
Pathology	5.86	0	0.0700	0.9300	0	13	1.509	12	0.723	11
pharmacy	5.73	0	0.1350	0.8650	0	14	1.317	16	0.582	14
Radiology-CT scan	5.54	0	0.2300	0.7700	0	15	1.341	15	0.564	15
maternity hospital	5.5	0	0.2500	0.7500	0	16	1.316	17	0.270	27
angiography	5.47	0	0.2650	0.7350	0	17	1.461	14	0.223	29
LDR	5.44	0	0.2800	0.7200	0	18	1.316	18	0.279	25
poisoned department	5.31	0	0.3450	0.6550	0	19	1.243	19	0.406	23
special surgery inpatient	5.26	0	0.3700	0.6300	0	20	1.210	20	0.499	20
Library	5.24	0	0.3800	0.6200	0	21	1.080	22	0.527	18
education	5.22	0	0.3900	0.6100	0	22		21	0.546	16
Pediatrics	5.18	0	0.4100	0.5900	0	23	1.066	24	0.809	9
administrative department	5.14	0	0.4300	0.5700	0	24	1.056	23	0.435	22
General Internal medicine	4.8	0	0.6000	0.4000	0	25	1.057	25	0.520	19
Internal medicine specialist	4.71	0	0.6450	0.3550	0	26	1.013	26	0.825	8
infectious	4.66	0	0.6700	0.3300	0	27	1.008	27	0.682	12
Property warehouse	4.49	0	0.7550	0.2450	0	28	0.941	28	0.356	24
emergency room	4.44	0	0.7800	0.2200	0	29	0.866	30	0.216	30
MRI	4.22	0	0.8900	0.1100	0	30	0.941	29	0.279	26

The Pearson correlation coefficient was estimated in order to compare the findings. The fire risk ratings for buildings and occupants obtained from the FRAME technique and the fire risk rating of the proposed method have a correlation coefficient of 62.1 % and 99.1 %, respectively. It reveals that the relationship between ranking fire risk for occupants and proposed method is positive and very strong.

However, as the fire risk magnitude acquired from the proposed approach for determining the fire risk of occupants is comparable to fire risk of occupants in the FRAME method, and the proposed method considers the factors related to occupant's fire safety.

Even though no significant ranking changes are seen between the FRAME approach and the current solution, it can be argued that the application of this integrated approach is novel in the field of fire safety assessment in healthcare centers. In addition, specific parameters are reviewed to assess the fire risk of healthcare centers, in this study.

As the second validation study, we actually carried out a sensitivity analysis by studying several departments in case study hospital. Based on these changes in the weight of the three parameters, we were able to estimate changes in the fire risk magnitude (FRM). This means that, as can be seen in Table 26, different values of RM have been obtained in IVN- AHP technique based on slightly changing three input parameters (PRL, ARL and PL). This demonstrates how sensitively the risk magnitude responds to adjustments in the input parameters' weight.

## 5 Discussion

The introduction of a novel approach for fire safety assessment in healthcare facilities through the utilization of IVN- AHP and FIS represents a groundbreaking advancement in addressing the complexities and uncertainties inherent in evaluating and managing fire risks within critical healthcare settings. By combining these advanced computational methodologies, this research initiative offers a comprehensive and flexible framework for enhancing the effectiveness and reliability of fire safety assessments, ultimately contributing to the overall resilience and safety of healthcare facilities.

One key aspect of this novel approach is the integration of IVN-AHP, which allows for a more nuanced and sophisticated evaluation of fire safety criteria by considering the interval-valued neutrosophic judgments and uncertainties associated with decision-making processes. This methodology enables healthcare facility managers and safety professionals to conduct a more detailed and comprehensive analysis of various fire safety factors, such as building layout, safety equipment distribution, and evacuation protocols, taking into account the imprecise and uncertain nature of the available information.

Moreover, the incorporation of a Fuzzy Inference System within the assessment framework enhances the modeling capabilities and decision-making processes by accommodating fuzzy inputs, incomplete data, and uncertainties in fire safety assessments. Through fuzzy logic principles and inference mechanisms, this system provides a flexible and adaptive approach to capturing the complex relationships and interactions among different fire safety parameters, enabling a more accurate and robust analysis of potential fire risks and mitigation strategies within healthcare facilities.

By introducing this approach for fire safety assessment in healthcare facilities, researchers and practitioners have the opportunity to revolutionize the way fire safety is evaluated and managed in critical healthcare settings. The integration of Interval Valued Neutrosophic AHP and Fuzzy Inference System not only improves the precision and reliability of fire risk assessments but also offers a more holistic and comprehensive approach to enhancing fire safety preparedness, response, and recovery measures within healthcare infrastructures. Risk assessment is facilitated, and findings are more accurate when all factors are systematically organized using a hierarchy and evaluated using expert opinions. Since the risk assessment precedes risk management applications, risk magnitude must be properly addressed to decide what to do afterward. Therefore, the results provided by the proposed approach can facilitate decision-making related to risk management strategies.

Overall, the adoption of this novel approach represents a significant step forward in advancing fire safety assessment practices in healthcare facilities, providing decision-makers with a powerful toolset to proactively identify, assess, and mitigate potential fire risks, ultimately contributing to the protection of lives, assets, and critical healthcare operations.

## 6 Conclusion

This work examines the issue of healthcare fire risk assessment. To identify the most significant risk factor for fire risk in healthcare facilities, seven experts are consulted, and their opinions were compiled using the fuzzy-Delphi method. Afterward**,** a novel IVN-AHP integrated FIS methodology was constructed and employed. The importance weights of each factor were calculated and then, PRL, ARL and PL parameters were scored as alternatives by IVN-AHP.

Subsequently, the risk magnitude of each ward or department in the healthcare building was estimated using FIS, and finally, its risk class was determined. All the steps mentioned above were performed for all departments in a hospital in Iran.

In the critical class, the utilities and kitchen departments were found to account for more than 50 %. Critical departments also included the operating room (46.5 %), laundry (45 %), NICU 1 (45 %), and 2 (33.5 %). However, nearly fifty percent fall into the major risk class. The utilities department in this hospital poses the greatest fire risk. According to the obtained results, none of the departments were categorized in a class with a minimal fire risk.

Therefore, it is important to use automatic protection systems and sufficient water supply systems in each department. The correlation coefficients between the fire risk ratings of the proposed method and the buildings and occupants derived using the FRAME technique were 99.1 % and 62.1 %, respectively. It reveals that the relationship between ranking fire risk for occupants and proposed method are positive and very strong.

Furthermore, contributions of this work to literature and practical application are outlined in the following list:

(1) The IVN-AHP, a neutrosophic multifactor decision-making technique, is optimized to fire risk assessment issue for healthcare facilities, (2) The most important factor regarding fire risk in healthcare are defined and categorized, (3) These factors and their sub-factors are evaluated by the proposed approach, and the importance weights of each factor are obtained, (4) The risk magnitude of each department in healthcare buildings is determined, and their risk class is determined based on the risk magnitude. (4) departments of a hospital in Iran are ranked according to fire risk, (5) The validity and reliability of the proposed risk assessment method is demonstrated in a real-case study, (5) The proposed risk assessment method is intended to be a useful fire risk assessment approach for hospitals, (7) It allows decision makers to make a rapid and effective assessment to address fire risks in healthcare. In addition, according to the statistical models performed on similar issues, incomplete information can be integrated into the decision-making process through expert evaluation. In this context, a practical evaluation method is presented.

## CRediT authorship contribution statement

**Samaneh Salari:** Writing – original draft, Software, Methodology, Formal analysis. **Ali Karimi:** Writing – original draft, Validation, Supervision, Investigation.

## Data availability statement

Data will be made available on request.

## Declaration of competing interest

The authors declare that they have no known competing financial interests or personal relationships that could have appeared to influence the work reported in this paper.
